# Extreme polygyny results in intersex differences in age-dependent survival of a highly dimorphic marine mammal

**DOI:** 10.1098/rsos.221635

**Published:** 2023-03-22

**Authors:** Sophia Volzke, Jaimie B. Cleeland, Mark A. Hindell, Stuart P. Corney, Simon J. Wotherspoon, Clive R. McMahon

**Affiliations:** ^1^ Institute for Marine and Antarctic Studies, University of Tasmania, Hobart, TAS 7005, Australia; ^2^ Centre for Marine Socioecology, University of Tasmania, Hobart, TAS 7005, Australia; ^3^ Australian Antarctic Partnership Program, University of Tasmania, Hobart, TAS 7005, Australia; ^4^ Australian Antarctic Division, Department of Agriculture, Water and the Environment, Kingston, TAS 7050, Australia; ^5^ IMOS Animal Tagging, Sydney Institute of Marine Science, Mosman, NSW 2088, Australia

**Keywords:** Cormack–Jolly–Seber model, demography, southern elephant seal, mark–recapture, *Mirounga leonina*

## Abstract

Developmental differences in vital rates are especially profound in polygamous mating systems. Southern elephant seals (*Mirounga leonina*) are highly dimorphic and extremely polygynous marine mammals. A demographic model, supported by long-term capture–mark–recapture records, investigated the influence of sex and age on survival in this species. The study revealed clear differences between female and male age-dependent survival rates. Overall juvenile survival estimates were stable around 80–85% for both sexes. However, male survival estimates were 5–10% lower than females in the same age classes until 8 years of age. At this point, male survival decreased rapidly to 50% ± 10% while female estimates remained constant at 80% ± 5%. Different energetic requirements could underpin intersex differences in adult survival. However, the species' strong sexual dimorphism diverges during early juvenile development when sex-specific survival rates were less distinct. Maximizing growth is especially advantageous for males, with size being a major determinant of breeding probability. Maturing males may employ a high-risk high-reward foraging strategy to compensate for extensive sexual selection pressures and sex-specific energetic needs. Our findings suggest sex-specific adult survival is a result of *in situ* ecological interactions and evolutionary specialization associated with being a highly polygynous marine predator.

## 1. Introduction

Studying extreme cases of long-lived polygynous and sexually dimorphic species informs the broader field of ecology by assigning weight to the contribution of evolutionary (genetic and phenotypic advantages), ecological (behavioural adaptation, intra- and interspecific competition and predation) and external (environmental) influences on population dynamics [1]. To analyse these complex interactions, modern demographic models are informed by advanced knowledge of the life history, developmental and reproductive biology of the study species [2]. Many mammals exhibit sexual size dimorphism, often with sexual selection pressures and resource limitation favouring larger males in polygynous mating systems [3–5]. These sexually mature males evolved distinct physiological characteristics (large body size, ornaments, reproductive organs, etc.) and behaviours (aggression, mating call, etc.) to increase their chances of gaining access to reproductive females [6]. Usually, these traits are gained or expressed as individuals mature, making them age-dependent. Thus, for many sexually dimorphic vertebrate species, intersex differences in survival rates become more apparent for adults, due to the discrete energetic requirements and behavioural traits that influence reproductive fitness, individual behaviour and vital rates [1].

**Figure 1.**
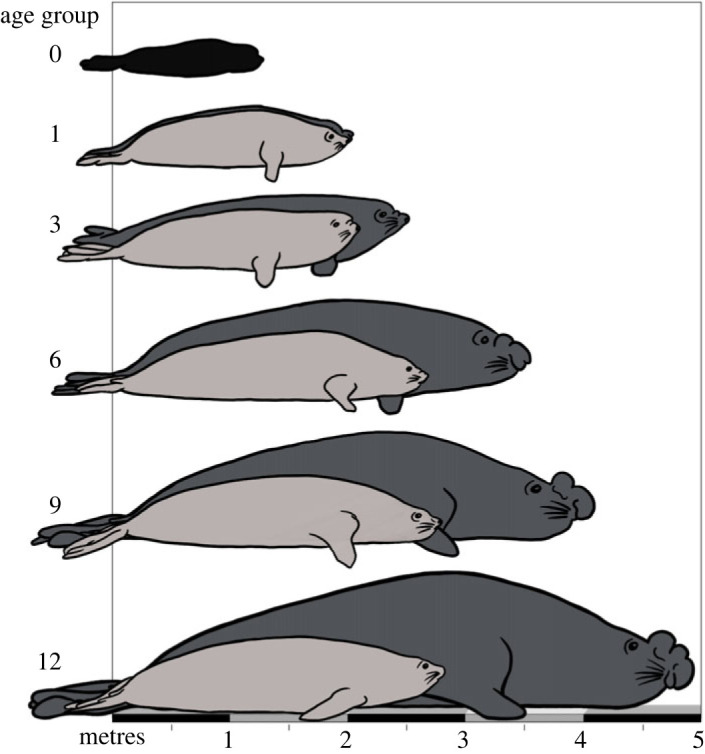
Average growth by age for male (dark grey) and female (light grey) southern elephant seals (*Mirounga leonina*). The comparable nose-to-tail lengths between sexes are illustrated by overlaying the average size of males and females in each age category. Age classes are divided into major developmental stages: black pup (0); yearling (1); juvenile (3) and mature adult females with sub-adult (6), subordinate (9) and socially mature (12) males. Mean length by age approximation inspired by Laws [7] and validated through records of known age individuals from Macquarie Island [8].

Adult male southern elephant seals reach nearly five times the body mass of females (figure 1; [9]). This species represents a model example of a polygynous mating system, which is mediated by sex-dependent developmental differences. Recent demographic research into southern elephant seal population dynamics (growth or decline) has concentrated on female survival and reproduction, quantifying important factors such as juvenile survival and fecundity [10–13]. Males do not contribute to parental care and so do not influence pup survival or recruitment [14]. However, the few (approx. 4%) adult males that do control breeding harems make a disproportionately large contribution to the gene pool of the next generation. Consequently, it is important to understand the vital rates of both sexes to determine the underlying evolutionary processes which influence the demography of such sexually dimorphic polygynous mammals [15]. Globally, demographic studies of the species illustrated a snapshot of early development by analysing sex-dependent differences in juvenile survival [16,17]. Other studies solely compared intersex differences in adult survival [18]. Very few have illustrated progressive changes to survival rates with age, and those that did were challenged by analytical limitations with the computing capacity of demographic models at the time of publication [19–21]. Advanced modern matrix population modelling techniques incorporate ontogenetic groupings to identify observed differences in survival probability across different developmental stages [22]. These approaches are frequently challenged by uncertainty in state assignment and many use proxies such as age to determine the ontogenetic status of individuals [23]. This research aimed to illustrate age-dependent survival for both male and female southern elephant seals using age- and time-dependent demographic models. Understanding these core survival parameters aids in assessing external influences on population viability, which enables projecting future threats to populations facing inevitable shifts in ecosystem composition.

Besides sex and age, developmental stage is one of the main distinguishing characteristics influencing southern elephant seal demography [24]. Studies report sex-specific adult survival, while juvenile survival is regarded as equal between sexes [25]. Juvenile males and females are similar in size and it is not until they reach biological maturity that strong sexual dimorphism is exhibited (figure 1) [26]. For juvenile seals from Macquarie Island, foraging ground segregation is related to age, rather than sex, with younger seals staying closer to their natal island, making shorter and more frequent trips than older juveniles [27]. But for southern elephant seals from Îles Kerguelen, habitat partitioning occurs before males and females diverge in body size [28]. It remains unclear whether sexual segregation in their foraging range is due to a difference in prey sources, intraspecific competition or physiological constraints [9]. Females forage across broad ranges of the Southern Ocean, but largely avoid sea ice [28,29]. By contrast, adult males concentrate their foraging effort in shallower waters [29,30]. These highly productive locations are frequented by other marine predators, such as orcas (*Orcinus orca*) and sleeper sharks (*Somniosus antarcticus*). Males may be employing a high-risk high-reward foraging strategy to attain a competitively large body size as quickly as possible [28]. Females in turn, maximize their lifetime reproductive output by using a less risky foraging strategy. This distinction would result in intersex differences in survival. Historic long-term capture–mark–recapture records from Macquarie Island present a great opportunity to test this theory.

We estimated age-specific survival rates for male and female southern elephant seals from Macquarie Island. A long-standing assumption that survival in this species is influenced by developmental (e.g. energetic cost of growth and reproduction) [8,21,31] and evolutionary (e.g. sexual selection, competition and predation) [28,32] constraints underpinned this approach. To investigate the effect of polygyny on demographic rates, we quantified the influence of sex and age on southern elephant seal survival and investigated a range of theories including if juvenile survival differs from adult survival in both sexes; if overall male survival is significantly lower than female survival; and lastly, in line with other populations, if intersex differences in survival become more apparent as individuals age and sexually mature. This research illustrates key demographic processes that influence survival in a highly polygynous marine mammal.

## 2. Methods

### 2.1. Study species

Southern elephant seals are large, capital breeding marine mammals. Figure 1 highlights the differences in growth between the sexes for different developmental stages. Most females make their first breeding attempt at 3–5 years old [12,24]. Males attain biological maturity at 6–7 years old, meaning they are physiologically capable of reproduction [33]. However, in order to make a successful mating attempt, they must spend another 4–5 years growing to competitive bull size [26]. We refer to biologically developed males which are not yet socially mature as sub-adult (ages 5–8) and subordinate (ages 9–12). A small number of males survive beyond these stages and grow large enough to control a harem of up to 100 females in the breeding season [26]. This adult sex ratio makes them one of the most polygynous of all mammals [9].

### 2.2. Data collection

At Macquarie Island (54°30′ S, 158°57′ E), 14 175 pups from seven cohorts (1993–1999) were marked by hot-iron branding shortly after weaning [34]. Branding does not negatively affect survival [35] and produces a lifetime individual mark that is easily read from a safe distance [36]. Until 2001, standardized surveys collected resights of marked individuals (i) daily on the main breeding site of the northern isthmus beaches, (ii) every 10 days on the northern third of the island, and (iii) once a month across the whole island [37]. After that, resights were collected ad hoc through alignment with other projects and population censuses up until 2015. Sex was recorded at weaning and confirmed through ongoing records. Only individuals of known sex were included in the analysis, resulting in a sample size of 6999 males and 7009 females. Individual resights were converted into an encounter history format by summarizing sightings annually (electronic supplementary material, appendix S2).

### 2.3. Demographic analysis

Data extraction and analyses were performed in R v. 4.2.1 [38] with RStudio 2022.07.01 [39]. Capture–mark–recapture data were analysed using the software MARK v. 9.0 [40] through RMark [41]. Various single-state demographic models were constructed to estimate survival and detection probabilities using Cormack–Jolly–Seber models and derivatives (electronic supplementary material, appendix S3). We first selected the most parsimonious model structure for detection probabilities (p) by running alternatives of the global (most saturated) model. These were tested for temporal and intersex differences by including an interaction effect (electronic supplementary material, appendix S3). Survival probabilities (Phi) were modelled with various combinations of three possible covariates: age, time and sex (table 1). We included uniform baseline models with constant detection, as well as single effect models and additive as well as multiplicative interaction terms. A global model with a three-way interaction influencing survival was also included. Models were compared by Akaike information criterion (AIC) [42] and ultimately ranked by quasi-AIC corrected (qAICc) [43] to incorporate a conservative overdispersion factor ĉ of 1.7 (electronic supplementary material, appendix S1). Goodness-of-fit tests were conducted on single-state encounter histories using Median-ĉ simulation in MARK ([40], electronic supplementary material, appendix S1).

**Table 1.** A combination of single-state age- and time-dependent demographic models were tested for intersex differences in detection (p) and survival (Phi) with package RMark [41]. Model selection table, organized by qAICc values in decreasing order. To demonstrate robustness, additional columns show rankings by AIC and AICc. The ‘order’ column indicates the order in which the models were ranked for each statistic. The ‘model’ column details the components and interaction terms tested in each model. The number of parameters is listed in the column labelled ‘npar’, as estimated by MARK [40], and adjusted for full rank by RMark.

model	npar	qAICc with ĉ = 1.7			AIC		AICc	
		order	qAICc	*Δ*qAICc	order	AIC	order	AICc
Phi(∼SEX × age) p(∼time × SEX)	88	1	68032.05	0.00	1	115530.7	1	115531.0
Phi(∼SEX + age) p(∼time × SEX)	67	2	68044.43	12.38	2	115581.4	2	115581.6
Phi(∼time + SEX) p(∼time × SEX)	67	3	68202.36	170.32	4	115849.9	4	115850.1
Phi(∼time × SEX) p(∼time × SEX)	88	4	68206.10	174.06	3	115826.6	3	115826.9
Phi(∼SEX) p(∼time × SEX)	46	5	68280.24	248.19	5	116011.8	5	116011.9
Phi(∼age) p(∼time × SEX)	66	6	68408.33	376.29	8	116201.4	8	116201.6
Phi(∼time) p(∼time × SEX)	66	7	68538.32	506.28	10	116422.4	10	116422.6
Phi(∼time × SEX × age) p(∼time × SEX)	592	8	68753.16	721.12	6	116024.5	6	116040.4
Phi(∼time × SEX × age) p(∼time + SEX)	571	9	68807.78	775.73	7	116148.6	7	116163.5
Phi(∼time × SEX × age) p(∼time)	570	10	68915.90	883.85	9	116333.9	9	116348.7
Phi(∼time × SEX × age) p(∼SEX)	550	11	70334.66	2302.62	11	118775.6	11	118789.3
Phi(∼time × SEX × age) p(∼1)	549	12	70481.29	2449.24	12	119026.3	12	119040.0

## 3. Results

### 3.1. Model selection

Goodness-of-fit testing indicated a small level of overdispersion, which was accounted for by incorporating a conservative ĉ = 1.7 (electronic supplementary material, appendix S1), using qAICc as the main statistic for model selection (table 1). The most parsimonious model included sex for both detection (p) and survival estimates (Phi). Additionally, the selected model included time-varying detection probabilities in addition to incorporating an age effect into survival estimates (table 1). These were included as a multiplicative interaction term, which varies survival with all ages ‘Phi(∼SEX × age)’ and detection across all years ‘p(∼time × SEX)’ (electronic supplementary material, appendix S3).

### 3.2. Demographic model

**Figure 2.**
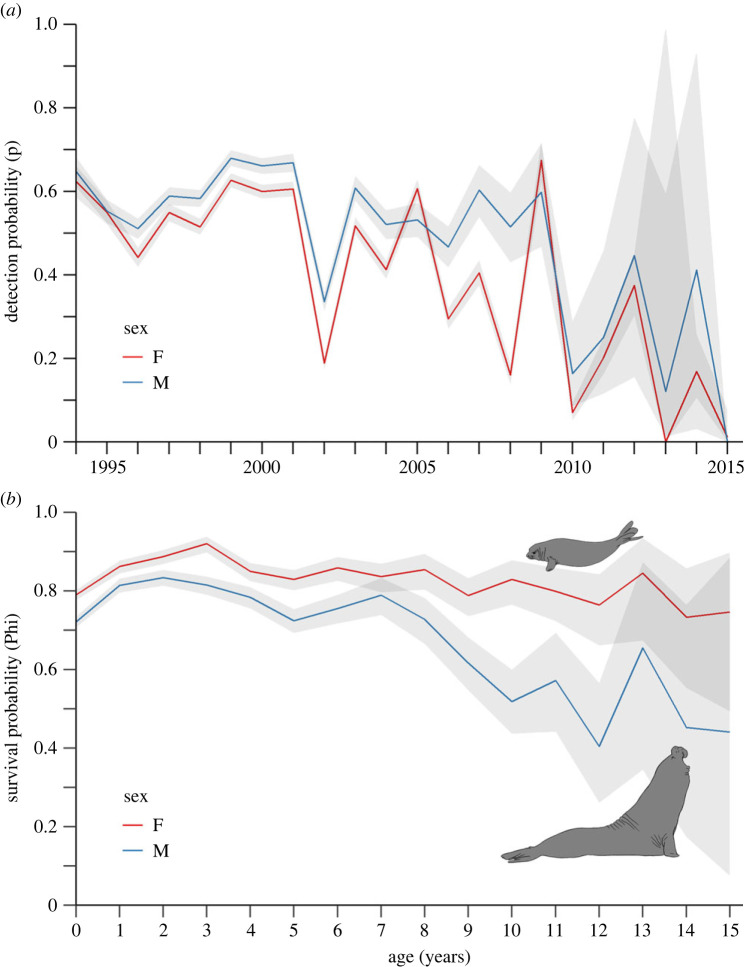
Estimated probabilities of detection (*a*) and survival (*b*) of a single-state demographic model constructed from comprehensive capture–mark–recapture histories of 14 008 southern elephant seals from Macquarie Island. Estimates are plotted separately for females (red) and males (blue) with corresponding 95% confidence intervals represented by grey error shades. The *x*-axis represents the relevant dependent variable of the most parsimonious model: time (calendar years) for detection probabilities (p) and age (years since first capture) for survival estimates (Phi). Probability estimates on the *y*-axes are plotted as decimal values from 0 to 1, equivalent to percentages from 0 to 100% respectively.

Figure 2 illustrates annual detection probabilities over the study period (*a*) and modelled probabilities of survival with age (*b*) for male and female southern elephant seals. Annual variation in detection accounted for varying resight effort over the study period. Throughout the active monitoring period (1993–2001), mean detectability of both male and female seals was evenly high (approx. 50% F and 60% M). During ad hoc sampling from 2001, sex-specific detection became more variable with high fluctuations between the sexes, before declining rapidly after 2009 (figure 2*a*).

Mean estimates of female survival (0.741 ± 0.077) were higher than male survival (0.637 ± 0.102) in all age classes (figure 2*b*). Young (ages 1–4) survival of both sexes followed a similar increasing trend. Female survival declined rapidly from age 3 (90%) to age 4 (85%), then remained steady with a very slight decreasing trend until age 12 (75%). However, the variance around these estimates also increased with age. The model results make it difficult to distinguish slightly decreasing adult female survival from steady 80% survival with greater variability. Male survival estimates were consistently lower than females in the same age classes until 8 years of age, after which they feature a sudden downturn from a steady 74% ± 5% to 50% ± 10% by age 10, a clear diversion from female adult survival estimates (80%). Modelled estimates become highly variable as sample size decreases, with mean male survival varying between 35% and 75% from ages 13 onwards (figure 2*b*). It is possible that a small sample of successful individuals growing much older than others drives the variability around modelled predictions close to 0–100% survival. Therefore, we decided to cut off age-dependent survival results at 15 years for both sexes.

## 4. Discussion

We quantified age-dependent survival between male and female southern elephant seals. Overall male survival was lower than female survival, as found previously on Macquarie [16,19] and Marion Island [21]. By incorporating age, we demonstrated a clear developmental difference in sex-dependent survival. Male survival was consistently 5–10% below females for juvenile ages (figure 2*b*). It should be noted that this model did not account for weaning mass, which is known to influence juvenile survival in this species [44]. In the first few weeks of development, male pups and weaners are heavier than females and so might be expected to have higher survival [7,45]. However, the energetic costs of raising male pups are higher and smaller females may terminate pregnancies to prioritize their own growth [46]. A covariate of weaning mass would be useful to further investigate intersex differences in juvenile survival that may not have been accounted for by these models.

Overall, seal detections were high during active monitoring (1993–2001) with greater variation between and within sexes post 2001 after organized searches were replaced by ad hoc sampling (figure 2*a*). Including data post 2001 was essential to determining which seals tagged in the early 1990s survived to adulthood. Later in the study, (2005–2010) detection of males was higher than females, probably because larger adult males were easier to see. Additionally, there may be greater chances of encountering a male during the period of ad hoc sightings because breeding adult males spend more time on beaches than females, as they arrive prior to the start of the breeding season [47]. Lastly, a steady decline in estimated detection probabilities for both sexes after 2009 was expected due to conclusion of active monitoring. At this point in time, no new individuals were being marked and the cohort of branded animals decreased due to age-related mortality. This contributed to increased variability around survival estimates of older ages.

Goodness-of-fit tests confirmed that age or developmental stage of individuals influenced the demographic rates of this species. The calculated overdispersion factor was well within acceptable range. We tested the robustness of the models by including multiple information criteria in the model selection (table 1; see electronic supplementary material, appendix S1.3: Analysis). Each criteria selected the same two top-ranked models, both of which included the same parameters in the same order. Because of these consistencies, the biological interpretation of this analysis is robust to a reasonable amount of potential overdispersion in the data.

Survival trends for young (ages 0–4) males and females were similar, increasing in the first 2–3 years of development, then slowly decreasing at a matching rate. Likewise, growth rates of juvenile males and young females matched initially, then diverged quickly after 7 years of development, when females are known to be fully mature [12] (figure 1). Developmental requirements and attempting to breed for the first time may contribute to the sharp decrease in female survival estimates by age 4 (figure 2*b*) [12]. Size and overall body condition is a major determinant of survival and reproductive age in maturing female southern elephant seals [12,48]. As capital breeders, females rely on energy stores to sustain themselves and to nurse their pups while fasting ashore during three weeks of terrestrial lactation [14]. Individuals in poor condition can skip a breeding season to conserve energy [11]. By contrast, males have a very low chance of contributing to future generations. A small proportion of high-quality males gain dominant breeding status and maintain high survival and reproductive success, even in future breeding seasons [49]. The remainder of mature males struggle to survive in this highly competitive breeding system. This effect is captured by the large amount of variability in survival estimates for adult males in our study (figure 2*b*). It should be noted that maturing subordinates may stay away from harems if they made an unsuccessful attempt at establishing dominance [33]. To account for this, we used a comprehensive suite of resights from whole island surveys to minimize potential missed counts. Male survival estimates decreased rapidly between the ages of 7–10, dropping nearly 25% in just 3 years to a mean of 50% by age 10 (figure 2*b*). During juvenile and sub-adult development, all accumulated resources are converted to growth (figure 1). Subordinate males become socially competitive for mating around 10–12 years old [33]. Additionally, maturing males shift their haul-out patterns to align with the early breeding season [19]. This behaviour requires large amounts of energy stores to sustain individuals while they are fasting ashore and fighting with other males for dominance over a harem. Therefore, an energetic need and competition for prey resources could be underpinning the decrease in survival at these key developmental stages [31,50]. In elephant seals, energetic costs of growth are tightly linked to reproductive success [51]. This is also the case for other well-studied polygamous mammals with male-dominant breeding systems and expressed sexual dimorphism. For example, in terrestrial ungulates such as fallow deer (*Dama dama*) [52] and bighorn sheep (*Ovis canadensis*) [53], dominant males monopolize mating events by having a larger body mass and greater horn or antler size.

Intersex differences in survival with age are probably the result of the complex social dynamics in this highly polygynous mating system. Many studies of southern elephant seals have reported a substantial difference in adult survival between the sexes [19]. Despite a 50/50 sex ratio at birth, adult males make up only 36% of the adult population [54]. For seals past their first year of life, mortality on land is negligible [25]. Even in the energetically expensive breeding season, the vast majority of females return to the ocean after weaning their pups [20]. Adult males can suffer serious injuries from competing with other bulls over mating access, but these fights rarely end in fatalities [20]. At-sea mortality is therefore regarded as the major cause of death, either by starvation or predation [20,25]. Outside the breeding season, elephant seals spend most of their life foraging at sea [19]. Tracking studies from the Kerguelen population found strong evidence for foraging habitat partitioning between adult male and female southern elephant seals [28]. Females frequented the open mid-ocean regions, while males preferentially foraged along the Antarctic continental shelf and the shallow plateau regions surrounding their natal island [28]. This behaviour was also observed in the Macquarie Island population, although at a limited sample size [29]. By contrast, young (ages 1–4) juvenile males and females from Macquarie Island visited similar areas, with age being the main determinant of their at-sea movements [27]. This strongly suggests an age, or developmental, component to sex-specific foraging behaviour in this species. Diet studies support this assertion, with age being the sole determinant of prey sources found in 1- to 4-year-old juveniles from Macquarie Island [55]. Resource partitioning between sexes becomes apparent for mature adults [56–58], which is attributed to diverging foraging preferences of adults [59].

In conclusion, the overall lower survival of males could be linked to the species' dimorphism and sex-specific energetic needs, which diverge early in the seal's life history [8]. Unsurprisingly, adult females survived longer than males on average. However, our study illustrated an increased mortality risk for socially maturing males later in life. Diverging intersex differences in survival became abruptly apparent for seals aged 9 years or older. This indicates that this species is subject to the pressures associated with being a highly polygynous and sexually dimorphic marine predator. To develop into a dominant breeding adult, maturing males must compete for limited prey resources to gain and sustain an extreme amount of body mass very fast. Simultaneously, socially maturing males must be able to fight with competing bulls and remain ashore for lengthy periods during the breeding season. To compensate for these high energetic demands, adult males employ riskier foraging strategies, placing them in areas where they may encounter other top predators [30]. Thus, maturing sub-adult and subordinate males are at greater risk of starvation and predation. Females on the other hand, maximize their lifetime reproductive output by following a wide-ranging pelagic foraging strategy, away from productive shelf waters [60]. Additionally, females can compensate for less successful feeding bouts by prolonging their foraging trips and returning later in the breeding season [19,45]. In extreme cases, females in poor body condition may skip a breeding season entirely, thereby conserving the extreme energetic cost of birthing, nurturing and weaning a pup [11]. Our findings suggest sex-specific adult survival is a result of *in situ* ecological interactions and evolutionary specialization associated with being a highly polygynous marine predator.

## Data Availability

Macquarie Island southern elephant seal capture–mark–recapture data are openly available in Dryad (https://doi.org/10.5061/dryad.zpc866t7f) [37] and have been previously published in the Australian Antarctic Data Centre [34]. The data and code supporting this article are provided in the electronic supplementary material [61].
